# Molecular Characterization and Pathogen-Responsive Expression of Galectin-8 in Starry Flounder (*Platichthys stellatus*): Preliminary Assessment of Leukocyte ROS Responses

**DOI:** 10.3390/ani16213534

**Published:** 2026-11-09

**Authors:** Chae-Yeong Ji, Ju-Won Kim, Min-Young Sohn, Ha-Jeong Son, Su Bhin Jeong, Hee Jeong Kong, Chan-Il Park

**Affiliations:** 1 Department of Marine Biology & Aquaculture, Gyeongsang National University, 2 Tongyeonghaeanro, Tongyeong 53064, Republic of Korea; aslhey123@naver.com (C.-Y.J.); subin8084@naver.com (S.B.J.); 2 Genetics and Breeding Research Center, National Institute of Fisheries Science, Geoje 53334, Republic of Korea; ogamzar@korea.kr (J.-W.K.); heejkong@korea.kr (H.J.K.); 3 Department of Institute of Marine Industry, Gyeongsang National University, 2 Tongyeonghaeanro, Tongyeong 53064, Republic of Korea; thisdancemoment@naver.com (M.-Y.S.); jemma9120@naver.com (H.-J.S.)

**Keywords:** galectin-8, reactive oxygen species (ROS), immune-associated activity, *Platichthys stellatus*, *Streptococcus parauberis*, viral hemorrhagic septicemia virus

## Abstract

Galectin-8 is a tandem-repeat member of the galectin family involved in cellular and immune-related processes in vertebrates, but its role in teleost fish remains poorly understood. In this study, galectin-8 (*PsGal8*) was identified and characterized in starry flounder, and its tissue distribution and expression following viral and bacterial challenges were investigated. The predicted PsGal8 protein contained two carbohydrate recognition domains connected by a linker peptide and possessed conserved carbohydrate-binding motifs characteristic of galectin-8. *PsGal8* transcripts were detected in all examined tissues, with the highest expression in the trunk kidney. Following the viral hemorrhagic septicemia virus challenge, significant changes in *PsGal8* expression were observed in the liver, intestine, and heart. The *Streptococcus parauberis* PH0710 challenge also modulated *PsGal8* expression, with significant upregulation in the spleen, liver, brain, and intestine at 7 days post-infection. In an exploratory in vitro assay, peripheral blood leukocytes exposed to a recombinant PsGal8-containing preparation showed increased reactive oxygen species fluorescence at concentrations of 5 μg/mL and above. However, the preparation was not fully homogeneous, and its endotoxin content was not quantified. This observation should be considered preliminary. Overall, these findings provide molecular and transcriptional evidence that *PsGal8* is responsive to viral and bacterial challenges in starry flounder.

## Simple Summary

Starry flounder (*Platichthys stellatus*) is an important aquaculture fish species in the Republic of Korea, but infectious diseases can cause serious health problems and economic losses. Understanding how this fish responds to infection may help improve future disease prevention strategies. Galectin-8 is a protein involved in immune regulation in vertebrates, but little is known about its role in fish. In this study, we identified galectin-8 in starry flounder and examined its characteristics and responses to viral and bacterial infections. Galectin-8 was present in various tissues and was most abundant in the trunk kidney, an important immune organ in fish. Its expression changed in several tissues following infection with viral hemorrhagic septicemia virus or the bacterium *Streptococcus parauberis*, suggesting that it responds differently depending on the infection model and stage. We also conducted a preliminary laboratory experiment to examine whether a recombinant PsGal8-containing preparation could influence immune cells. Overall, our findings provide new information on galectin-8 in starry flounder and contribute to a better understanding of immune responses to infectious diseases in cultured fish.

## 1. Introduction

Galectins are a family of β-galactoside-binding lectins characterized by conserved carbohydrate recognition domains (CRDs). They are widely distributed in vertebrates and are involved in various biological processes, including cell adhesion, apoptosis, inflammation, and immune regulation [1]. Based on the organization of their CRDs, galectins are classified into three groups: prototype, chimera-type, and tandem-repeat galectins [2]. Prototype galectins contain one CRD and can form homodimers; chimera-type galectins contain one CRD and an N-terminal domain; and tandem-repeat galectins contain two CRDs linked by a peptide [1].

Galectin-8 is a tandem-repeat galectin composed of two distinct CRDs linked by a peptide [3]. The two CRDs of galectin-8 recognize different glycan structures. The N-terminal CRD preferentially binds sulfated and sialylated galactose-containing oligosaccharides, whereas the C-terminal CRD mainly recognizes non-sialylated glycans [2]. These structural features allow galectin-8 to interact with various glycoconjugates. In mammalian cells, galectin-8 has been associated with cytokine regulation and reactive oxygen species (ROS) production in neutrophils [4,5,6].

In teleost fish, galectin-8 has been reported in several species, including Nile tilapia (*Oreochromis niloticus*), black rockfish (*Sebastes schlegelii*), and olive flounder (*Paralichthys olivaceus*). These studies showed that galectin-8 is expressed in various tissues and is regulated after immune stimulation or pathogen challenge [7,8,9]. In addition, galectin-8 or galectin-8-like proteins have been associated with antibacterial activity, respiratory burst activity, and immune-related gene regulation in fish [3,10].

Starry flounder (*Platichthys stellatus*) is a cold-water marine flatfish. The importance of the starry flounder aquaculture industry has grown as a result of the need for an alternative species to replace olive flounder aquaculture in South Korea, which has been plagued by problems such as high mortality rates and unstable market conditions. As a substitute species, starry flounder production is on the rise [11]. Whilst production is increasing for this reason, the industry remains under threat from infectious diseases. Previous studies in starry flounder have reported infection by viral and bacterial agents, including megalocytivirus and *Streptococcus parauberis* [12,13]. However, the molecular characteristics and immune-associated responses of galectin-8 in starry flounder remain unclear.

Although galectin-8 has been characterized in several teleost species, its molecular features and expression patterns have not been investigated in starry flounder. Therefore, the present study aimed to identify and molecularly characterize galectin-8 from *P. stellatus* (*PsGal8*), determine its basal expression profile in different tissues, and examine its transcriptional responses following VHSV and *S. parauberis* PH0710 challenges. In addition, a preliminary in vitro assay was performed to examine ROS responses in peripheral blood leukocytes following exposure to a recombinant PsGal8-containing preparation. These data provide baseline information for further investigation of the biological roles of galectin-8 in the immune system of starry flounder.

## 2. Materials and Methods

### 2.1. Gene Identification and Sequence Analysis

To obtain the complete cDNA sequence of *P. stellatus* galectin-8, a set of gene-specific primers was designed to target the 5′ and 3′ ends of the gene (Table 1). These primers were then used in a polymerase chain reaction (PCR) to amplify the desired DNA sequence. *P. stellatus* galectin-8 (*PsGal8*) was obtained from a previously generated NGS-based transcriptome dataset [14]. PCR was performed using ExPrime Taq Premix (2×; Genetbio, Daejeon, South Korea) under the following cycling conditions: initial denaturation at 95 °C for 5 min, followed by 40 cycles of denaturation at 95 °C for 30 s, annealing at 58 °C for 30 s, and extension at 72 °C for 1 min, with a final extension at 72 °C for 10 min. The PCR products were separated on 1.2% agarose gels containing 0.01% SafeView Classic (abm, Richmond, BC, Canada), and bands of the expected size were excised and purified using the QIAquick Gel Extraction Kit (Qiagen, Hilden, Germany).

The objective of the present study was to determine the sequence of the PCR products and the amino acid sequence of the proteins. To this end, the previous research was reviewed, and the cloning method was employed [15]. In summary, the purified PCR products were ligated using the pGEM-T Easy vector (Madison, WI, USA), and the *Escherichia coli* JM109 competent cells were subjected to heat shock. Transformants were selected by blue–white screening on Luria–Bertani (LB) agar plates containing ampicillin, IPTG, and X-gal. The positive colonies were subsequently cultivated at 37 °C for 12 h in LB liquid medium. The plasmids were then extracted using the Hybrid-Q Plasmid Rapidprep Kit (GeneAll, Seoul, South Korea), and Sanger sequencing was performed by BIONEER (Daejeon, South Korea).

Amino acid sequence analysis and phylogenetic analysis of the *PsGal8* cDNA sequence were performed by referring to the analytical methods reported in previous studies [15]. The cDNA sequence of galectin-8 was analyzed using GENETYX ver. 8.0 (SDC Software Development, Japan) to identify its homologous sequences. The Galectin-8 homologous sequences were identified using the BLASTX program from the National Center for Biotechnology Information (NCBI) database (http://blast.ncbi.nlm.nih.gov/Blast.cgi, 4 January 2026). The conserved motifs and functional domains were predicted using the PROSITE (http://www.expasy.org/tools/scanprosite/, 4 January 2026) and SMART (http://smart.embl-heidelberg.de/, 4 January 2026) programs, respectively. The signal peptide of Gal8 was predicted using SignalP 6.0 (https://services.healthtech.dtu.dk/services/SignalP-6.0/, 4 January 2026). The multiple sequence alignment (MSA) was performed using ClustalX2, and the resulting alignment was refined using BioEdit version 7.7.1.0. The phylogenetic relationships were calculated using the ClustalW alignment, and the neighbor-joining method in MEGA 11.0 was used to construct the evolutionary tree. This method was selected to ensure the reliability of the results, and 1000 bootstrap replicates were analyzed to calculate the confidence levels. The nucleotide sequence of *PsGal8* was deposited in GenBank under accession number PZ506901.

### 2.2. Tissue Collection for Gene Expression Analysis

Healthy *P. stellatus* (n = 100; mean total length: 21.5 ± 1.3 cm; mean body weight: 128.7 ± 18.2 g) were obtained from a commercial farm (Pohang, South Korea). Fish were acclimated in laboratory tanks at 22 ± 1 °C, salinity 31–33 ppt, pH 7.6–7.9, and dissolved oxygen above 6.0 mg/L for two weeks under monitored conditions. Before the experiment, the fish were fed a commercial diet (Jeil Feed Co., Republic of Korea; 42% crude protein, 8% crude lipid) at 1.5% of body weight per day. Also, three individuals were randomly selected for clinical examinations to confirm their health status. All procedures involving *P. stellatus* were performed in accordance with the experimental protocol approved by the Institutional Animal Care and Use Committee (IACUC) of Gyeongsang National University, College of Marine Science (Approval No. GNU-241104-E0206; 4 November 2024).

To isolate samples, the fish were anesthetized and euthanized by immersion in seawater containing an overdose of benzocaine (Sigma-Aldrich, St. Louis, MO, USA; 200 mg/L). For tissue distribution analysis, 14 tissues were sampled: brain, eye, gills, head kidney, trunk kidney, heart, liver, intestine, muscle, skin, stomach, spleen, red blood cells (RBCs), and peripheral blood leukocytes (PBLs). Blood was collected from the caudal vein using heparinized syringes and separated into PBLs and RBCs by the Percoll density gradient method, as reported in previous studies [16,17].

The pathogens used in the experiments were provided by the National Institute of Fisheries Science (Busan, South Korea). For time-course analysis after immune stimulation, fish were intraperitoneally injected with either *S. parauberis* PH0710 (1 × 10^3^ CFU/fish) or viral hemorrhagic septicemia virus (VHSV, 1 × 10^6^ TCID_50_/fish) suspended in 100 µL PBS. Following injection, fish challenged with *S. parauberis* PH0710 were maintained at 25 °C, whereas VHSV-challenged fish were maintained at 17 °C. Fish injected with 100 μL of sterile PBS were used as the 0 h control group. The pathogen challenge experiments were conducted for seven days in a static-water system with complete daily water replacement. Throughout the experimental period, salinity was maintained at 32–33 ppt, pH at 7.5–7.8, and dissolved oxygen at approximately 5.9–6.1 mg/L. Three random biological replicates (n = 3) were sampled at 1 and 12 h post-injection (hpi) and at 1, 3, 5, and 7 days post-injection (dpi). Tissues (brain, gills, head kidney, heart, liver, intestine, and spleen) were collected aseptically and stored at −80 °C.

### 2.3. Total RNA Extraction and cDNA Synthesis from Tissues

Total RNA was extracted from collected tissues using RNAiso Plus (TaKaRa, Kusatsu, Japan), followed by additional purification using PCI (phenol–chloroform–isoamyl alcohol, 25:24:1). Genomic DNA was removed using Recombinant DNase I (TaKaRa, Kusatsu, Japan), followed by an additional PCI extraction step for further purification of the RNA samples, including the removal of residual proteins and other contaminants. RNA samples were precipitated with isopropanol, washed with 75% ethanol, and resuspended in DEPC-treated water. RNA concentration and purity were evaluated using a NanoVue spectrophotometer (GE Healthcare, Little Chalfont, UK). cDNA synthesis was performed using the PrimeScript™ 1st Strand cDNA Synthesis Kit (Takara, Kusatsu, Japan) following the manufacturer’s protocol.

### 2.4. Quantitative Real-Time PCR from Tissues

To analyze the expression level of *PsGal8*, TB Green Premix Ex Taq™ (TaKaRa, Kusatsu, Japan) was used, and the composition for each reaction was as follows: 12.5 µL of 2× premix, 1 µL each of forward and reverse primers, 1 µL of cDNA, and sterile distilled water. The qRT-PCR was performed on a Thermal Cycler Dice Real-Time System III (TaKaRa) with the following conditions: 50 °C for 4 min, 95 °C for 10 min, followed by 45 cycles at 95 °C for 20 s and 60 °C for 1 min. Using a previously reported set of primers, the elongation factor 1-alpha (EF-1α) was utilized as an internal reference gene, whilst the qRT-PCR primers for *PsGal8* were newly designed in this study based on the cDNA sequence obtained [14] (Table 1). Gene expression levels were normalized to EF-1α using the 2^−ΔΔCt^ method [18]. The amplification efficiency and specificity of the PsGal8 primer pair were validated using standard curve and melting curve analyses (Figure S1). The corresponding primer sequences and amplification efficiency parameters are provided in Table S1.

### 2.5. Recombinant PsGal8-Containing Preparation Synthesis and Western Blotting

Specific primers were designed to amplify the coding region of *PsGal8* with restriction enzyme recognition sites for *Bam*HI and *Not*I. The forward primer contained a *Bam*HI site, and the reverse primer contained a *Not*I site (Table 1). PCR amplification was performed under the same cycling conditions described in Section 2.1. PCR products were verified by agarose gel electrophoresis and purified using the QIAquick Gel Extraction Kit (Qiagen, Hilden, Germany). The purified amplicon and the pBIVT2 expression vector containing a 6 × His tag were digested with *Bam*HI and *Not*I at 37 °C for 2 h and ligated using T4 DNA ligase (Promega, Madison, WI, USA). The ligation mixture was transformed into *E. coli* JM109 competent cells, and the inserted sequence was confirmed by Sanger sequencing. A recombinant PsGal8-containing preparation was produced using a cell-free protein synthesis system (AccuRapid™ Protein Synthesis Kit, Bioneer, Daejeon, South Korea) according to the manufacturer’s protocol. Briefly, plasmid DNA including the *PsGal8* sequence was mixed with the *E. coli*-derived extract and master mix, and the reaction mixture was incubated at 30 °C for 3 h. The recombinant protein was subsequently enriched using Ni-NTA magnetic beads supplied with the AccuRapid™ Protein Synthesis Kit according to the manufacturer’s protocol. The Ni-NTA magnetic beads selectively bind to the 6 × His tag of recombinant PsGal8 through affinity interactions between immobilized nickel ions (Ni^2+^) and histidine residues, allowing the separation of His-tagged recombinant PsGal8 from other components of the cell-free reaction mixture. The bound protein fraction was subsequently recovered according to the manufacturer’s instructions. The resulting protein fraction was examined by 15% SDS-PAGE, and the protein concentration of the recovered fraction was determined using the Bradford method with the Bio-Rad Protein Assay Kit (Bio-Rad, Hercules, CA, USA). Because additional protein bands were present in the Ni-NTA-enriched fraction, the preparation is hereafter referred to as the recombinant PsGal8-containing preparation rather than as a homogeneous purified protein preparation.

Western blotting was performed to confirm the presence of the His-tagged recombinant PsGal8-containing preparation in the Ni-NTA-enriched protein fraction. The recombinant protein preparation was separated by 15% SDS-PAGE and transferred onto a nitrocellulose membrane using an iBlot™ Gel Transfer System (Invitrogen, Carlsbad, CA, USA). The membrane was blocked with Western BLoT Blocking Buffer (Protein Free; Takara, Kusatsu, Japan) at 4 °C for 12 h and washed three times with TBS-T containing 0.05% Tween-20. The membrane was then incubated with a mouse 6 × His tag monoclonal antibody (HIS.H8; Invitrogen, Carlsbad, CA, USA) diluted 1:2000 in Western BLoT Immuno Booster 1 (Takara, Kusatsu, Japan) for 2 h at room temperature. After three washes with TBS-T, the membrane was incubated with AP-conjugated anti-mouse IgG (Promega, Madison, WI, USA) diluted 1:2000 in TBS-T for 1 h at 37 °C. Following washing, immunoreactive bands were visualized using an alkaline phosphatase substrate (Promega, Madison, WI, USA) for 10 min according to the manufacturer’s instructions.

### 2.6. ROS Assay

Peripheral blood leukocytes (PBLs) were isolated from unchallenged starry flounder from the same stock described in Section 2.2 using a Percoll gradient according to a previously described method [17], with minor modifications. Briefly, 4 mL of 53% Percoll solution was placed in a 15 mL tube, and heparinized blood was collected from the caudal vein using a sterile syringe. The collected blood was mixed with Leibovitz’s L-15 medium (Sigma-Aldrich, St. Louis, MO, USA), and 4 mL of the suspension was carefully layered onto the Percoll solution. The sample was centrifuged at 400× *g* for 20 min, and leukocytes located between the Percoll layer and the medium were carefully collected using a pipette. The collected leukocytes were transferred to a 50 mL tube containing phosphate-buffered saline (PBS), centrifuged at 400× *g* for 10 min, and resuspended in PBS. The cell suspension was filtered through a 40 μm cell strainer, and the number of leukocytes was adjusted to 1 × 10^5^ cells/well. For assessment of ROS responses, PBLs were seeded into black flat-bottom 96-well plates and exposed to the recombinant PsGal8-containing preparation at final protein concentrations of 0.5, 1, 5, 10, 20, 40, and 80 μg/mL for 1 h at 15 °C. Hydrogen peroxide (100 μM) was included as a positive control.

Following treatment, 2′,7′-dichlorofluorescin diacetate (DCFH-DA; Sigma-Aldrich, St. Louis, MO, USA) was added to a final concentration of 10 μM, and cells were incubated for an additional 20 min in the dark at 15 °C. Fluorescence intensity was measured at excitation and emission wavelengths of 485 and 530 nm, respectively, using a microplate reader (Infinite M Plex, Tecan, Männedorf, Switzerland). ROS-associated fluorescence was expressed relative to that of the untreated control group. The assay was used as an exploratory assessment of leukocyte oxidative responses following exposure to the recombinant PsGal8-containing preparation.

### 2.7. Statistical Analysis

All data are presented as the mean ± standard deviation (SD) from three independent biological samples. Statistical analyses were performed using GraphPad Prism 11.0. Relative *PsGal8* mRNA expression levels were calculated using the 2^−ΔΔCt^ method, with EF-1α as the internal reference gene. For tissue distribution analysis, muscle was used as the reference tissue, and statistical differences between muscle and the other tissues were evaluated using one-way analysis of variance (ANOVA) followed by Dunnett’s multiple-comparison test. For pathogen challenge experiments, gene expression was analyzed separately for each tissue, with expression levels at each sampling time compared to the corresponding PBS-injected 0 h control group using one-way ANOVA followed by Dunnett’s multiple-comparison test. The VHSV and *S. parauberis* PH0710 challenge experiments were analyzed independently. For ROS analysis, differences between each treatment group and the untreated control were assessed using one-way ANOVA followed by Dunnett’s multiple-comparison test (n = 3, three individual fish). Significance levels were denoted as * *p* < 0.05, ** *p* < 0.01, *** *p* < 0.001 and **** *p* < 0.0001.

## 3. Results

### 3.1. Molecular Features of PsGal8

The coding sequence of *PsGal8* was 936 bp in length and encoded a predicted protein of 311 amino acids. The calculated molecular weight and theoretical isoelectric point were 33.93 kDa and 8.55, respectively. The nucleotide sequence was deposited in GenBank under accession number PZ506901.

**Figure 1:**
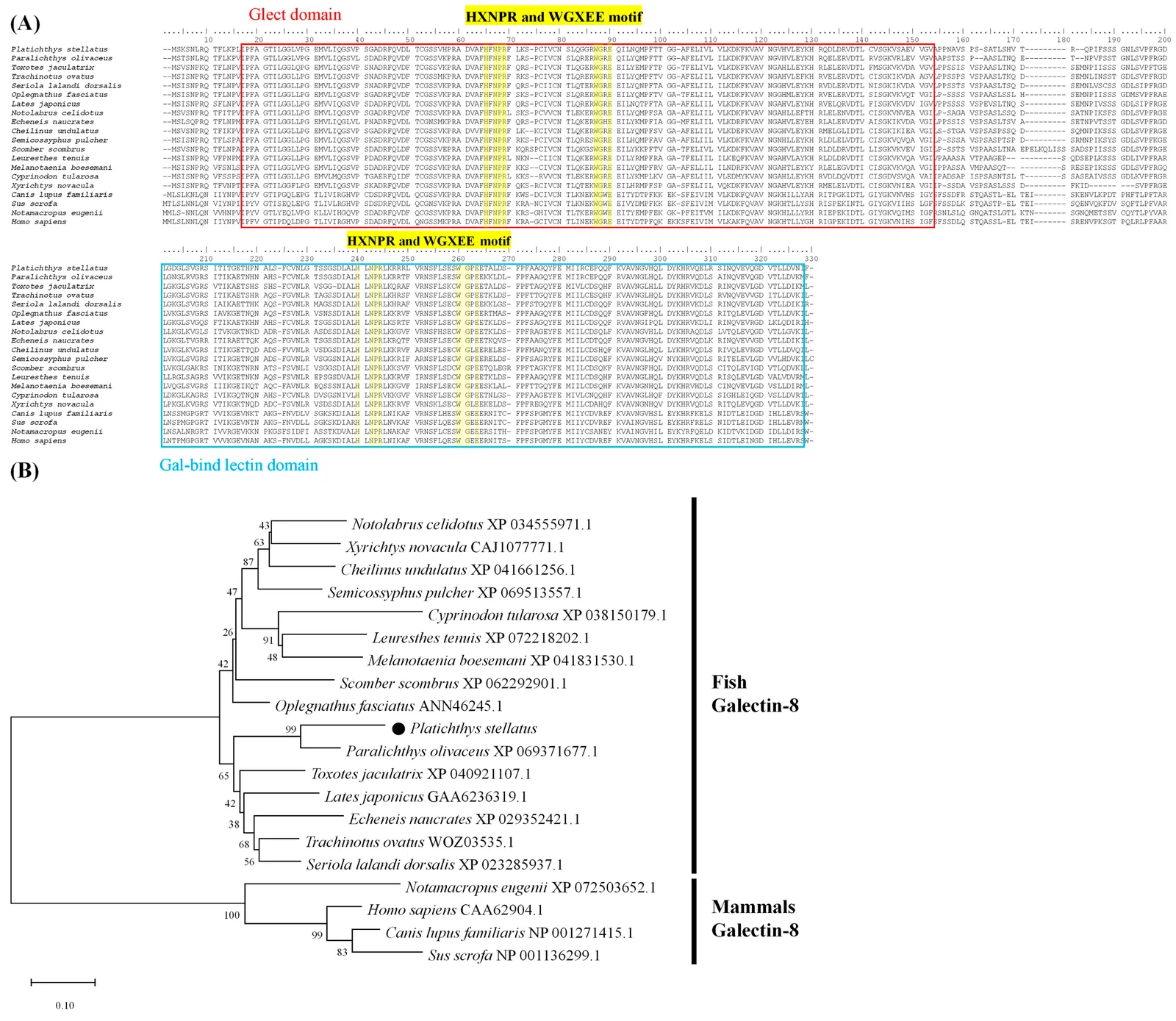
(**A**) Multiple alignment of galectin-8 amino acid sequences from *Platichthys stellatus* and other species. The carbohydrate recognition domains (CRDs) are indicated by red and blue boxes. Conserved residues involved in glycan binding are indicated by a yellow highlight. NCBI accession numbers of galectin-8 sequences used for alignment are as follows: *Paralichthys olivaceus* (XP_069371677.1); *Toxotes jaculatrix* (XP_040921107.1); *Trachinotus ovatus* (WOZ03535.1); *Seriola lalandi dorsalis* (XP_023285937.1); *Oplegnathus fasciatus* (ANN46245.1); *Lates japonicus* (GAA6236319.1); *Notolabrus celidotus* (XP_034555971.1); *Echeneis naucrates* (XP_029352421.1); *Cheilinus undulatus* (XP_041661256.1); *Semicossyphus pulcher* (XP_069513557.1); *Scomber scombrus* (XP_062292901.1); *Leuresthes tenuis* (XP_072218202.1); *Melanotaenia boesemani* (XP_041831530.1); *Cyprinodon tularosa* (XP_038150179.1); *Xyrichtys novacula* (CAJ1077771.1); *Canis lupus familiaris* (NP_001271415.1); *Sus scrofa* (NP_001136299.1); *Notamacropus eugenii* (XP_072503652.1); *Homo sapiens* (CAA62904.1). (**B**) Neighbor-joining phylogenetic tree of galectin-8 amino acid sequences constructed using MEGA 11.0. Bootstrap values were calculated from 1000 replicates. The scale bar indicates the evolutionary distance.

Sequence analysis identified two carbohydrate recognition domains (CRDs), located at amino acid residues 15–150 and 185–310, which were separated by a linker region. Both CRDs contained conserved carbohydrate-binding motifs characteristic of galectins, including HXNPR- and WGXEE-related motifs (Figure 1A). No conventional signal peptide was predicted.

Multiple sequence alignment showed that *PsGal8* shared conserved residues with galectin-8 homologues from other vertebrates. Most teleost galectin-8 sequences examined in this study contained HLNPR and WGXEET motifs, whereas the corresponding region in several mammalian galectin-8 sequences contained WGXEER. Among the fish sequences analyzed, *PsGal8* showed the highest amino acid sequence identity with *Paralichthys olivaceus* galectin-8 (82.96%) and the lowest identity with that of *Cyprinodon tularosa* (65.71%). Phylogenetic analysis placed *PsGal8* within the teleost galectin-8 cluster, distinct from the mammalian galectin-8 group (Figure 1B).

### 3.2. Tissue Distribution and Pathogen-Responsive Expression of PsGal8

**Figure 2:**
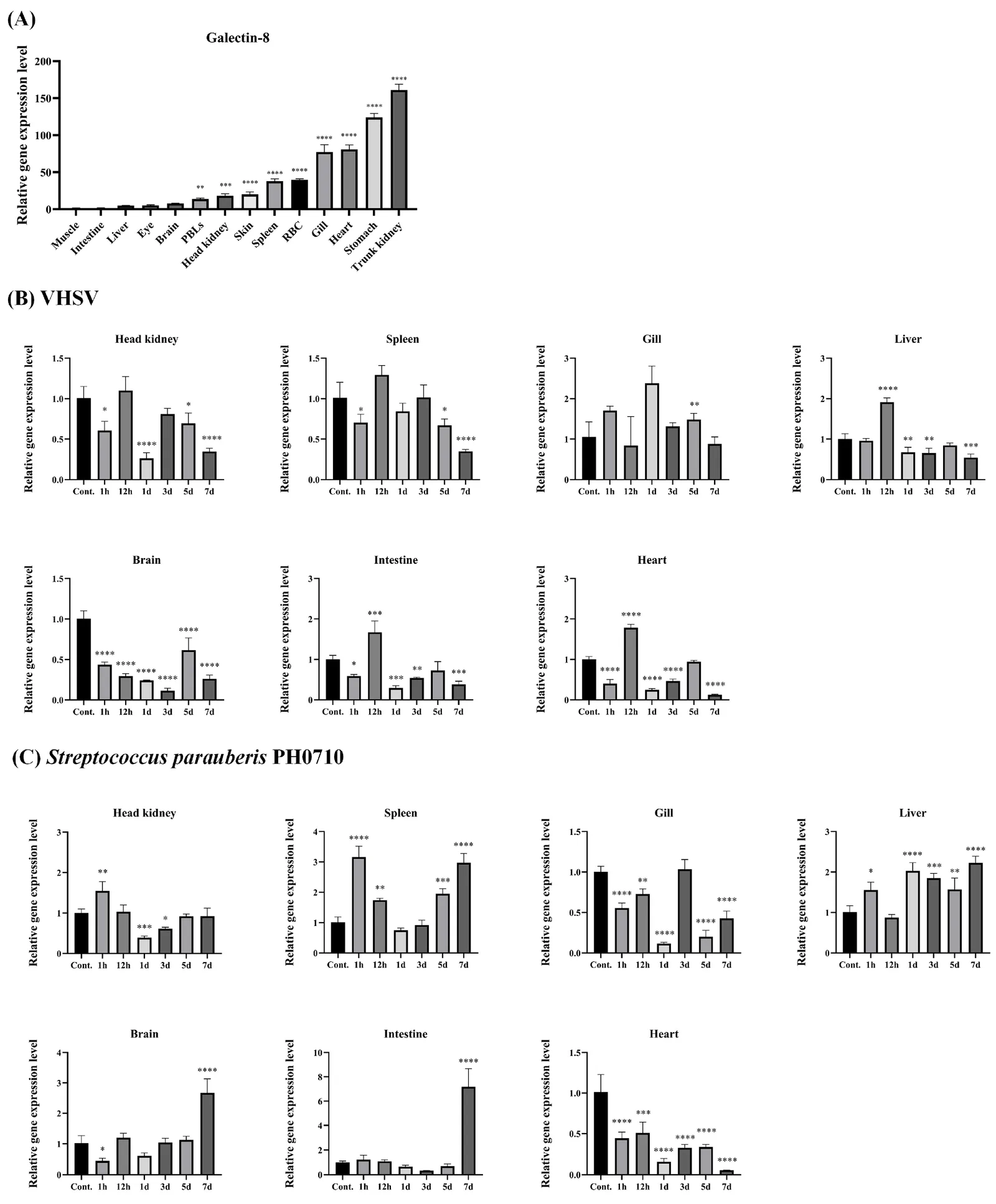
(**A**) Gene expression analysis of the galectin-8 gene by quantitative real-time PCR in various tissues of healthy *P. stellatus*. The EF-1α gene was used to normalize the qRT-PCR results. Expression levels are shown as fold change relative to the value in muscle. Statistical significance was determined using one-way ANOVA followed by Dunnett’s multiple-comparison test, with muscle as the control group. Comparisons among non-control groups were not performed. Asterisks indicate significant differences (* *p* < 0.05, ** *p* < 0.01, *** *p* < 0.001, **** *p* < 0.0001) versus muscle. All data are presented as the mean ± SD from three independent biological samples. The qRT-PCR analysis of the galectin-8 mRNA expression profile in the brain, gills, head kidney, heart, liver, intestine, and spleen of *P. stellatus* after infection with viral hemorrhagic septicemia virus (**B**) and *Streptococcus parauberis* PH0710 (**C**). Gene expression levels are presented as the mean ± SD (n = 3). Statistical significance was determined separately for each tissue using one-way ANOVA followed by Dunnett’s multiple-comparison test, with the corresponding PBS-injected 0 h group as the control. Asterisks indicate significant differences (* *p* < 0.05, ** *p* < 0.01, *** *p* < 0.001, **** *p* < 0.0001) versus the PBS-injected 0 h control group. Comparisons among non-control groups were not performed.

*PsGal8* transcripts were detected in all 14 tissues examined from healthy starry flounder. The highest mean fold change relative to muscle was observed in the trunk kidney (161-fold), followed by the stomach (124-fold), heart (80-fold), and gills (77-fold), all significantly higher than muscle (**** *p* < 0.0001) (Figure 2A).

Following the VHSV challenge, *PsGal8* expression varied among tissues and sampling times. Significant increases were observed in the liver, intestine, and heart at 12 h post-injection. In several tissues, expression subsequently decreased at later sampling points, although tissue-specific differences were evident throughout the experimental period (Figure 2B).

Following the *S. parauberis* PH0710 challenge, *PsGal8* expression was also modulated in a tissue-dependent manner. Significant upregulation was detected in the spleen, liver, brain, and intestine at 7 days post-injection. In the brain, *PsGal8* expression remained below the 0 h control level at several earlier sampling points before increasing at 7 days post-injection. In contrast, expression in the gills tended to remain lower than the control level at most sampling points (Figure 2C).

Because the VHSV and *S. parauberis* challenge experiments were conducted under different temperature conditions, their temporal expression profiles were considered independently rather than directly compared.

### 3.3. Detection of Recombinant PsGal8-Containing Preparation

**Figure 3:**
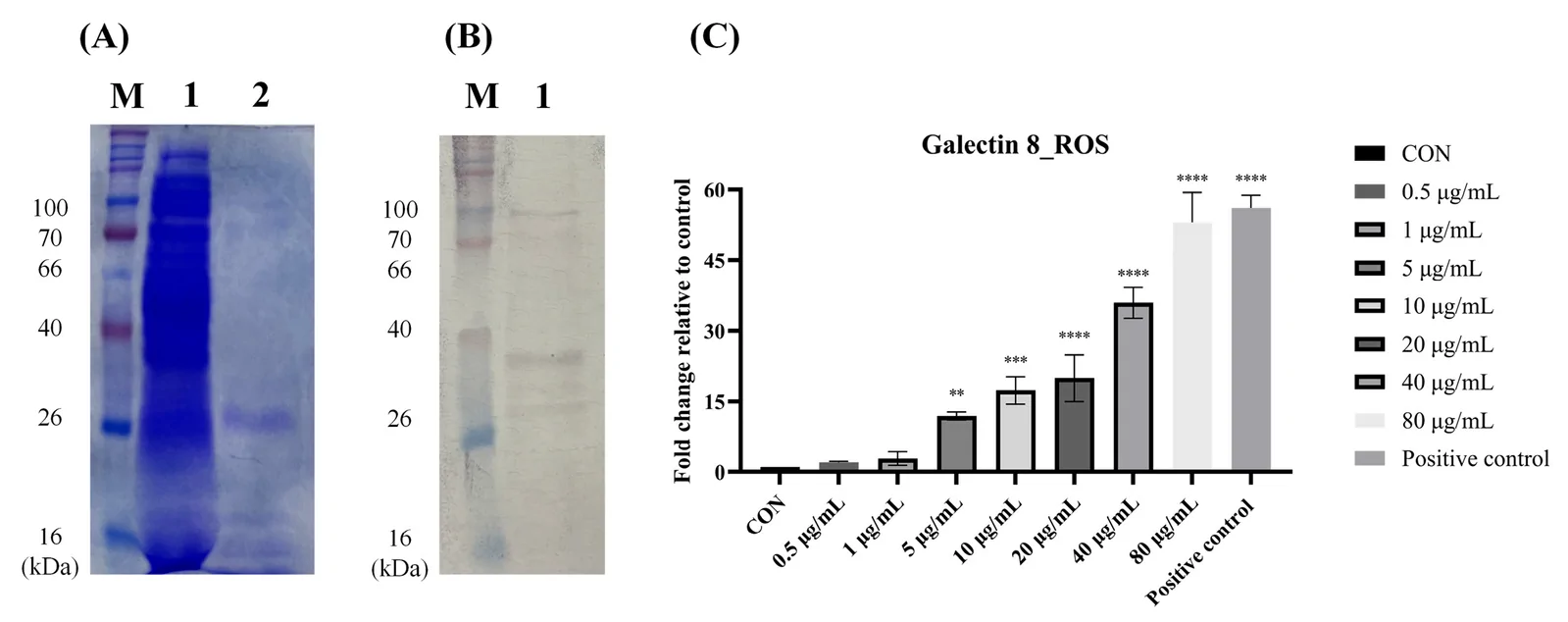
(**A**) SDS-PAGE analysis of recombinant PsGal8-containing preparation produced using the pBIVT2 cell-free expression system. A prominent protein band corresponding to the predicted molecular weight of PsGal8 (~35 kDa). Additional protein bands were also present in the Ni-NTA-enriched fraction. Lane M, molecular weight marker; Lane 1, unprocessed recombinant protein synthesis mixture; Lane 2, Ni-NTA-enriched recombinant PsGal8-containing preparation. (**B**) Western blot analysis using an anti-His antibody. A His-reactive band was detected at approximately 35 kDa, consistent with the predicted size of the His-tagged fusion protein. Lane M, molecular weight marker; Lane 1, Ni-NTA-enriched recombinant PsGal8-containing preparation. (**C**) ROS-associated fluorescence in peripheral blood leukocytes following treatment with the recombinant PsGal8-containing preparation. Cells were exposed to protein concentrations ranging from 0.5 to 80 μg/mL, and H_2_O_2_ was used as a positive control. ROS-associated fluorescence was measured using DCFH-DA. Data are presented as the mean ± SD (n = 3, three individual fish). Statistical significance was determined using one-way ANOVA followed by Dunnett’s multiple-comparison test, with the untreated group as the control. Asterisks indicate significant differences compared with the untreated control (** *p* < 0.01, *** *p* < 0.001, **** *p* < 0.0001).

A recombinant PsGal8-containing preparation was produced using the pBIVT2-based cell-free expression system and enriched using Ni-NTA affinity purification. The theoretical molecular weight was recalculated based on a structure with a verified sequence, including a vector-derived C-terminal AAA linker and 6 × His tag; the predicted molecular weight of the fusion protein is 320 amino acids (~35.0 kDa), which compares to the 33.93 kDa of native PsGal8. SDS-PAGE analysis of the Ni-NTA-enriched fraction showed a prominent protein band at approximately 35 kDa, corresponding to the predicted molecular weight of PsGal8, together with additional protein bands (Figure 3A).

Western blot analysis using an anti-His antibody detected a His-reactive band at approximately 35 kDa, consistent with the predicted size of the His-tagged fusion protein (Figure 3B). The original uncropped Western blot is provided in Figure S2, and the corresponding densitometric reading of the recombinant PsGal8 band is presented in Table S2.

### 3.4. ROS Responses of Peripheral Blood Leukocytes Following Treatment with the Recombinant PsGal8-Containing Preparation

The ROS response of peripheral blood leukocytes was examined following exposure to different concentrations of the recombinant PsGal8-containing preparation. No significant difference in ROS fluorescence was observed at concentrations up to 1 μg/mL compared with the untreated control. ROS fluorescence increased from 5 μg/mL onward and showed a concentration-related increase across the higher concentrations tested (Figure 3C).

## 4. Discussion

In the present study, galectin-8 was identified and molecularly characterized in starry flounder. *PsGal8* displayed the typical structural organization of a tandem-repeat galectin, including two CRDs separated by a linker region, and its transcript was detected in a broad range of tissues. *PsGal8* expression was also modulated following VHSV and *S. parauberis* PH0710 challenges. In addition, an exploratory in vitro experiment showed increased ROS fluorescence in peripheral blood leukocytes exposed to a recombinant PsGal8-containing preparation. Together, these findings provide initial molecular and transcriptional information on galectin-8 in starry flounder and a basis for subsequent functional studies.

Galectin-8 belongs to the tandem-repeat class of galectins and contains two structurally distinct CRDs in its N- and C-terminal regions [2,19]. *PsGal8* showed this characteristic domain organization and contained conserved HXNPR- and WGXEE-related carbohydrate-binding motifs. These conserved residues are associated with carbohydrate recognition by galectins [2,20]. Most teleost galectin-8 sequences analyzed in the present study contained a WGXEET motif, whereas several mammalian sequences contained WGXEER. A similar WGXEET motif has been reported in fish galectin-8, and variation within carbohydrate-binding regions has been suggested to be associated with differences in glycan recognition among galectin-8 homologues [2,10]. However, the functional significance of this sequence variation in *PsGal8* remains to be experimentally determined. In this study, it was found that the *PsGal8* sequence lacks a signal peptide. Previous research has shown that galectin-8 in *Cynoglossus semilaevis* is present in micro-vesicles [10]. These findings suggest the possibility that galectin-8 may be secreted via a non-classical pathway.

Furthermore, consistent with previous observations regarding other galectins [21], it is highly likely that *PsGal8* is synthesized in the cytoplasm rather than via the conventional endoplasmic reticulum–Golgi secretory pathway. These findings suggest that the predicted N-glycosylation sequence in *PsGal8* may not undergo typical N-linked glycosylation under physiological conditions [22]. Therefore, the absence of eukaryotic-type N-linked glycosylation in a bacterial cell-free expression system does not necessarily imply a significant difference from the native protein in this regard. Previous studies have shown that no signal peptide is observed and that recombinant galectin-8 retains its carbohydrate-binding capacity when expressed in an *E. coli* system [10]. Nevertheless, the glycosylation status of native *PsGal8* remains unknown, and further experimental validation, such as carbohydrate-dependent activity, is required to determine whether differences in post-translational modifications between native and recombinant *PsGal8* affect its biological activity.

*PsGal8* also contains a linker region of approximately 34 amino acids between its two CRDs. The linker or hinge region is a characteristic structural component of tandem-repeat galectin-8 and shows considerable variation in length and sequence among species and isoforms [2,9]. Such variation may influence the relative orientation and flexibility of the N- and C-terminal CRDs [2]. The linker lengths observed among the fish galectin-8 sequences examined in the present analysis further indicate substantial interspecific variation. Nevertheless, because the carbohydrate-binding properties of *PsGal8* were not directly examined in this study, any functional implications of the CRD or linker sequence characteristics remain speculative.

*PsGal8* transcripts were detected in all examined tissues, with particularly high expression in the trunk kidney. Broad tissue distribution of galectin-8 has also been reported in other teleost species, although relative expression levels differ among species and organs [7,8,9]. In olive flounder, galectin-8 expression has been reported in multiple tissues, including immune-related organs such as the kidney and spleen [9]. The teleost kidney is a major lympho-hematopoietic organ and contains diverse immune cell populations involved in both innate and adaptive immune responses [23]. Relatively high *PsGal8* expression was also observed in the stomach, heart, and gills. The gills and gastrointestinal tract represent important interfaces between the host and the external environment and contribute to mucosal immune defense in teleost fish [24]. Thus, the broad tissue distribution of *PsGal8* suggests that it may participate in multiple physiological and immune-associated processes rather than being restricted to a single immune organ.

Following the VHSV challenge, significant modulation of *PsGal8* expression was detected in several tissues, with increased transcription observed particularly in the liver, intestine, and heart at 12 h post-injection. Galectins can recognize host- and pathogen-associated glycoconjugates and have been implicated in various host responses to infection [25]. In teleost fish, galectin-8 and galectin-8-like molecules have also been reported to exhibit altered transcription following immune stimulation or pathogen exposure [7,8,9]. More recently, a galectin-8-like isoform in red-lip mullet was reported to be involved in immune responses associated with viral challenge [26]. The present transcriptional data therefore indicate that *PsGal8* is responsive to VHSV challenge. However, increased expression following viral infection does not demonstrate antiviral activity per se. No viral replication, viral clearance, neutralization, or survival assay was performed to directly examine the antiviral function of *PsGal8*. Accordingly, the present results should be interpreted as evidence of VHSV-responsive transcription rather than direct antiviral activity.

*PsGal8* expression was also altered following *S. parauberis* PH0710 challenge, with significant upregulation detected in the spleen, liver, brain, and intestine at 7 days post-injection. Regulation of galectin-8 expression following bacterial challenge has been reported in several teleost species [7,8,9]. In addition, recombinant galectin-8 or galectin-8-like proteins from some fish species have been reported to interact with bacteria or exhibit antibacterial-associated activities [3,10,27,28]. Also, galectin-8 variants have been associated with disease resistance [29]. In red-spotted grouper, recombinant galectin-8 was reported to agglutinate several Gram-positive and Gram-negative bacteria, including *S. parauberis* [27]. Experimental *S. parauberis* infection in starry flounder has also been shown to induce innate immune responses in multiple tissues [13], and temporal changes in immune-related gene expression following challenge with the same bacterial strain have previously been reported in this species [14]. Thus, the expression pattern observed in the present study supports an association between *PsGal8* transcription and the host response to *S. parauberis* infection. Nevertheless, direct antibacterial properties of *PsGal8* were not evaluated in the present study. Therefore, the observed transcriptional changes should not be interpreted as demonstrating bactericidal, bacteriostatic, or pathogen-clearance activity.

The transcriptional responses observed following VHSV and *S. parauberis* challenges should also be considered in the context of the experimental design. The VHSV-challenged fish were maintained at 17 °C, whereas the *S. parauberis*-challenged fish were maintained at 25 °C to accommodate the respective infection models. VHSV infection dynamics and pathogenicity in flatfish are strongly influenced by temperature conditions [30,31], while physiological responses of starry flounder can also vary according to water temperature [32]. Therefore, differences in temporal expression profiles between the two challenge experiments cannot be attributed solely to pathogen type. Consequently, the present results should not be interpreted as evidence that *PsGal8* inherently mediates an early antiviral response and a late antibacterial response. Instead, expression patterns following each pathogen challenge should be interpreted independently within their respective experimental conditions.

ROS are important mediators of cellular signaling and participate in inflammatory and antimicrobial responses in vertebrate immune cells [33]. Mammalian galectin-8 has been reported to modulate neutrophil function and stimulate oxidative responses [5], and galectin-8-like proteins in teleost fish have also been associated with respiratory burst activity and regulation of immune responses [3]. In the present study, treatment of starry flounder PBLs with the recombinant PsGal8-containing preparation was associated with increased DCFH-DA fluorescence beginning at 5 μg/mL. These observations are broadly consistent with previous reports linking galectin-8-related molecules with leukocyte oxidative activity [3,5]. In addition, in the case of galectin, it has been reported that galectin-3 activates NADPH oxidase in neutrophils within exudates but does not activate it in peripheral blood neutrophils [34], whereas another study demonstrated that galectin-3 inhibits the production of reactive oxygen species (ROS) induced by *Staphylococcus aureus* during phagocytosis [35]. Since these findings were obtained from mammalian neutrophils and galectin-3, they cannot be directly generalized to PsGal8 in teleost leukocytes; however, they raise the possibility that galectin-mediated oxidative reactions may vary depending on cell type, activation status, and stimulus.

However, the ROS experiment has several limitations that require cautious interpretation. First, although Ni-NTA enrichment produced a prominent band corresponding to the predicted molecular weight of PsGal8, and Western blotting confirmed the presence of His-tagged recombinant PsGal8-containing preparation, additional protein bands remained in the preparation. Thus, this preparation cannot be considered homogeneous. Second, a recombinant PsGal8-containing preparation was produced using an *E. coli*-based cell-free expression system, and endotoxin content was not quantified or experimentally controlled. Therefore, the potential contribution of components other than the recombinant PsGal8-containing preparation to the observed ROS response cannot be completely excluded. Consequently, the present ROS data do not establish a direct causal effect of PsGal8 on leukocyte oxidative activity.

The concentrations used for the ROS assay should also be interpreted cautiously. Significant changes became apparent at concentrations of 5 μg/mL and above, and the strongest response was observed at 80 μg/mL. However, endogenous recombinant PsGal8-containing preparation concentrations in the plasma or tissues of starry flounder have not been determined. Therefore, particularly the higher recombinant protein concentrations used in vitro cannot presently be considered physiologically representative. To further clarify the potential immunomodulatory function of PsGal8, additional experiments using highly purified recombinant protein preparations with appropriate endotoxin controls are necessary. Future studies using more highly purified and appropriately controlled recombinant protein preparations will be necessary to determine whether PsGal8 directly regulates leukocyte ROS production and to establish the concentration range at which such effects occur.

Despite these limitations, the present study expands current information on the galectin family in teleost fish by providing the first molecular characterization of *PsGal8* in starry flounder. Previous studies have demonstrated diverse molecular and immune-associated characteristics of galectin-8 homologues in Nile tilapia, black rockfish, olive flounder, and other teleost species [3,7,8,9,10,24,25,26]. In this context, the conserved tandem-repeat structure, broad tissue distribution, and transcriptional modulation of *PsGal8* following viral and bacterial challenges extend current knowledge of galectin-8 in marine flatfish. The preliminary ROS observation further provides a basis for future investigation, although it should not be regarded as definitive evidence of immunostimulatory, antibacterial, or antiviral activity.

## 5. Conclusions

In this study, galectin-8 was identified and molecularly characterized for the first time in starry flounder (*Platichthys stellatus*). *PsGal8* exhibited the conserved structural features of tandem-repeat galectins and was broadly expressed in various tissues, with the highest transcript level observed in the trunk kidney. The tissue-dependent modulation of *PsGal8* expression following VHSV and *Streptococcus parauberis* PH0710 challenges indicates that *PsGal8* is responsive to both viral and bacterial infections. In addition, increased ROS-associated fluorescence was observed in peripheral blood leukocytes exposed to the recombinant PsGal8-containing preparation; however, this finding should be regarded as preliminary because of limitations associated with protein purity and unquantified endotoxin content. Overall, this study provides fundamental molecular and transcriptional information on *PsGal8* and supports its possible association with immune responses in starry flounder. Further studies using highly purified and appropriately controlled recombinant PsGal8 are required to clarify its direct effects on leukocyte function and its specific roles in host defense against viral and bacterial pathogens.

## Figures and Tables

**Table 1:** Primers used in this study.

Usage	Primer Name	Primer Sequence (5′–3′)	Amplicon Size (bp)	Annealing Temperature (°C)	GenBank Accession Number
CDS sequence, verification	*Ps*Gal8*-cds-F*	ATGTCGAAATCAAACCTGAG	936	58	PZ506901
	*Ps*Gal8*-cds-R*	TCAGAAGATGTTGACGTCGA			
qRT-PCR (control)	*Ps*EF-1α*-F*	GTGGCAAGTCCACCACCA	135	60	MT044472.1
	*Ps*EF-1α*-R*	GCTTGTCCAGCACCCAGG			
qRT-PCR	*Ps*Gal8*-F*	GCAGGACAGTACTTTGAGAT	89	60	PZ506901
	*Ps*Gal8*-R*	TGTTTGTAGTCTAGCTGGTG			
Recombinant protein	*rPs*Gal8*-F*	GGATCCCATGTCGAAATCAAACCTGAG	955	58	
	*rPs*Gal8*-R*	GCGGCCGCGAAGATGTTGACGTCGAGCA			

Underline: Restriction enzyme sites for *Bam*HI (GGATCC) and *Not*I (GCGGCCGC) are underlined.

## Data Availability

The data presented in this study are available from the corresponding author upon reasonable request. The nucleotide sequence of PsGal8 has been deposited in GenBank under accession number PZ506901.

## Supplementary Materials

The following supporting information can be downloaded at: https://www.mdpi.com/article/10.3390/ani16213534/s1. Figure S1: Validation of the amplification efficiency and specificity of the PsGal8 primer pair; Figure S2: Original uncropped Western blot corresponding to Figure 3B; Table S1: Primer sequences and amplification efficiency parameters of the PsGal8 primer pair; Table S2: Densitometric reading of the recombinant PsGal8 band shown in Figure S1.

## Abbreviations

The following abbreviations are used in this manuscript:

Gal8	Galectin-8
PsGal8	*Platichthys stellatus* galectin-8
CRD	Carbohydrate recognition domain
VHSV	Viral hemorrhagic septicemia virus
ROS	Reactive oxygen species
PBL	Peripheral blood leukocyte
