# Supplementary material for: Molecular Characterization and Pathogen-Responsive Expression of Galectin-8 in Starry Flounder (Platichthys stellatus): Preliminary Assessment of Leukocyte ROS Responses
Source: Animals (Basel). 2026 Nov 9;16(21):3534. doi: 10.3390/ani16213534 (PMC13761821; doi:10.3390/ani16213534)
Supplement: Supplementary file 1 [file animals-16-03534-s001.zip › animals-4587988-supplementary.pdf]

Figure S1. Validation of the amplification efficiency and specificity of the PsGal8 primer pair. (A) Amplification curves of seven serial two-fold dilutions of cDNA (1- to 64-fold), each analyzed in triplicate. (B) Standard curve generated by plotting Ct values against the logarithm of the relative initial template quantity (1 = undiluted cDNA). The amplification efficiency was 91.1%, with a slope of −3.556 and a coefficient of determination (R^2) of 0.996. (C) Melting curve analysis showing a single sharp peak at approximately 82–83 °C.

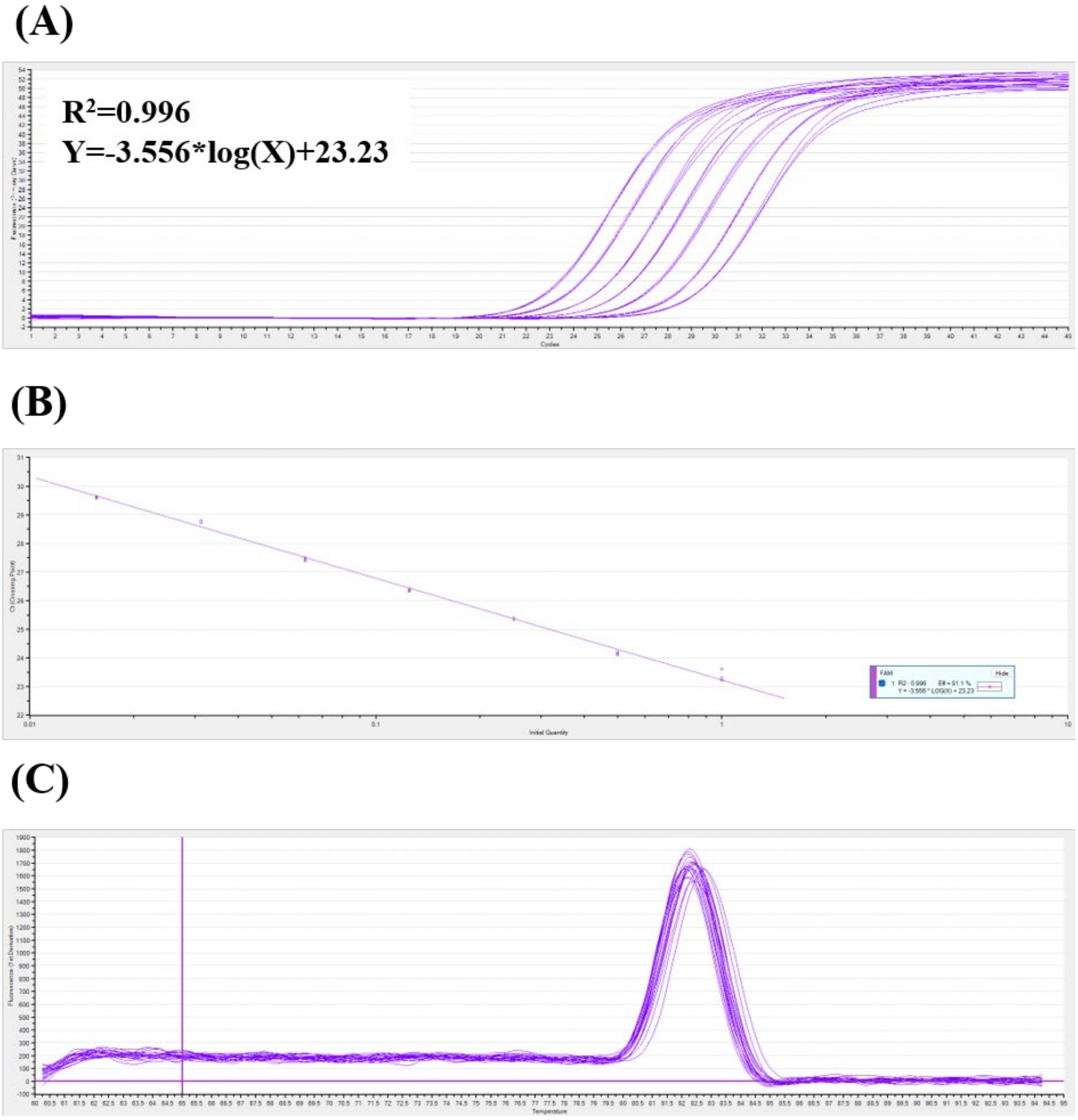

Table S1. *Primer sequences and amplification efficiency parameters of the PsGal8 primer pair.*

Usage	Primer	Primer Sequence (5′-3′)	Base pair	Efficiency (%)	R^2
Target gene	PsGal8 (F)	GCAGGACAGTACTTTGAGAT	89	91.1	0.996
	PsGal8 (R)	TGTTTGTAGTCTAGCTGGTG			

Figure S2. Original uncropped Western blot corresponding to Figure 3B. The uncropped Western blot image shows the molecular weight marker (M) and recombinant PsGal8 (lane 1) detected using an anti-His antibody. The immunoreactive band corresponding to recombinant PsGal8 was detected at approximately 33.9 kDa.

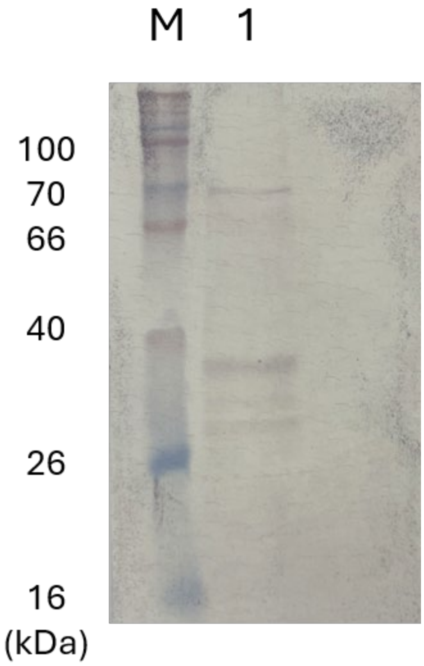

Table S2. Densitometric reading of the recombinant PsGal8 band shown in Figure S1.

**Lane**	**Protein**	**Approximate MW**	**Background-corrected integrated density (a.u.)**
1	Recombinant PsGal8	~33.9 kDa	5.72×10^3

*Densitometry was performed on the original digital photograph using local background subtraction. Because the Western blot was performed for qualitative confirmation of recombinant PsGal8 and contained only a single sample lane, a relative intensity ratio between experimental groups was not calculated.
